# Applications of CT-based radiomics for the prediction of immune checkpoint markers and immunotherapeutic outcomes in non-small cell lung cancer

**DOI:** 10.3389/fimmu.2024.1434171

**Published:** 2024-08-22

**Authors:** Jie Zheng, Shuang Xu, Guoyu Wang, Yiming Shi

**Affiliations:** ^1^ Department of Radiology, Taizhou Central Hospital, Taizhou University Hospital, Taizhou, Zhejiang, China; ^2^ Department of Radiology, Redcliffe Hospital, The University of Queensland, Redcliffe, QLD, Australia

**Keywords:** non-small cell lung cancer, radiomics, CT, immunotherapy, immune checkpoint

## Abstract

In recent years, there has been significant research interest in the field of immunotherapy for non-small cell lung cancer (NSCLC) within the academic community. Given the observed variations in individual responses, despite similarities in histopathologic type, immunohistochemical index, TNM stage, or mutation status, the identification of a reliable biomarker for early prediction of therapeutic responses is of utmost importance. Conventional medical imaging techniques primarily focus on macroscopic tumor monitoring, which may no longer adequately fulfill the requirements of clinical diagnosis and treatment. CT (computerized tomography) or PEF/CT-based radiomics has the potential to investigate the molecular-level biological attributes of tumors, such as PD-1/PD-L1 expression and tumor mutation burden, which offers a novel approach to assess the effectiveness of immunotherapy and forecast patient prognosis. The utilization of cutting-edge radiological imaging techniques, including radiomics, PET/CT, machine learning, and artificial intelligence, demonstrates significant potential in predicting diagnosis, treatment response, immunosuppressive characteristics, and immune-related adverse events. The current review highlights that CT scan-based radiomics is a reliable and feasible way to predict the benefits of immunotherapy in patients with advanced NSCLC.

## Introduction

According to the Cancer Statistics 2023 report in the United States, lung cancer is the second most prevalent type of malignancy in both males and females, representing 12% of new male cancer diagnoses and 13% of new female cancer diagnoses (1). The estimated new cases of lung cancer are predicted at 238,340 in the USA. Besides, lung cancer contributes the greatest number of deaths in men as well as in women, accounting for the same proportion of 21% of estimated deaths of all (1). A variety of risk factors contribute to lung cancer. These factors can be broadly categorized as nonmodifiable or modifiable. The former includes age, gender, family history, and genetics (2). The latter includes cigarette smoking, secondhand smoke, radon exposure, occupational exposure, air pollution, previous lung diseases, personal history of cancer, diet, and immunosuppression (2–4). Two types of lung cancer are generally recognized: small cell lung cancer (SCLC, accounting for 10 to 15% of all lung cancer) and non-small cell lung cancer (NSCLC, accounting for 85% of lung cancer cases) (5). NSCLC can be further divided into many subtypes, including adenocarcinoma, squamous cell carcinoma, large cell carcinoma, adenosquamous carcinoma, and other rare types (6). Patients with lung cancer have a survival rate of 10-20% 5 years after diagnosis due to this disease is typically diagnosed in the middle or late stages in most countries (1). Treatments for NSCLC include surgery, radiotherapy, chemotherapy, immunotherapy, and combined therapies, depending on the type and stage of the cancer (7). In advanced NSCLC, chemotherapy is typically applied as an adjuvant treatment. Chemotherapy for long periods is underwhelming due to intrinsic and extrinsic resistance, low target selectivity, and adverse effects. A progressive treatment may trigger multidrug resistance in lung cancer cells, resulting in failure to respond to chemotherapy. In the case of early-stage NSCLC, surgical resection is the most appropriate form of treatment. In advanced cases, partial cancer cells will be killed using radiotherapy, chemotherapy, or immunotherapy, which can reduce the morbidity and mortality of the sufferers (8). Since there are great individual differences among NSCLC patients, surgery supplemented by chemotherapy often fails to achieve a satisfactory therapeutic effect.

In recent years, immunotherapy has become a hot therapeutic method for NSCLC. Mounting evidence shows that the usage of immune checkpoint inhibitors (ICIs) in the treatment of NSCLC patients can effectively prolong the survival of patients with advanced and metastatic NSCLC (9). Many kinds of programmed death-1 (PD-1) and its ligand (PD-L1) monoclonal antibodies have been approved and applied to the treatment of advanced or locally advanced NSCLC (10), opening up a new horizon in NSCLC treatment. Radiological imaging has always been an effective tool for monitoring NSCLC, including tumor progression and therapeutic efficacy (11). With advanced imaging technology, it is possible to qualitatively or quantitatively analyze how patients’ medical images are correlated with immune checkpoints and to evaluate their prognosis as well as the efficacy of immunotherapy (12).

As compared to the traditional imaging analyses, CT-based radiomics allows for the extraction of a large number of quantitative imaging features from CT scans, which can capture tumor heterogeneity, serve as potential biomarkers for predicting treatment response to immunotherapy, stratify the patients into different risk groups, and assess the immunotherapy response. Therefore, CT-based radiomics may accurately predict treatment outcomes and guide therapeutic strategies, such as identifying factors contributing to treatment resistance or adverse events and assisting clinicians in selecting the most appropriate treatment options and monitoring patient response over time. This article reviews the application of radiological imaging in NSCLC immunotherapy.

## Molecular mechanisms of immunotherapy for NSCLC

Immunotherapy now comprises classical and ICI therapy. Classical immunotherapy involves active (tumor vaccines) and passive immunity (natural and artificial). ICIs have gained prominence for their efficacy in advanced NSCLC treatment (13, 14). Immunotherapy works by activating the immune system to recognize and eliminate tumor cells. However, tumors can evade immune surveillance, promoting proliferation and disease progression. Tumor cells suppress CD8+ T-cells, hindering the immune response. Regulatory T cells also impact tumor immunity by down-regulating CD8. ICIs block this immune suppression by targeting checkpoint markers like CTLA-4. CTLA-4 inhibitors, like ipilimumab and tremelimumab, were previously underutilized due to adverse effects but are now essential for NSCLC treatment (15, 16).

PD-1/PD-L1 antibodies revolutionize NSCLC treatment, offering superior antitumor effects over chemotherapy. NCCN guidelines recommend two years of immunotherapy maintenance for first-line patients and until disease progression for second-line patients. High PD-1/PD-L1 expression indicates greater immunotherapy benefit, alongside tumor mutation burden and initial tumor volume (17). Challenges persist in PD-L1 detection due to spatial and temporal heterogeneity, lack of standardized detection methods, and controversy over tumor mutational burden (TMB) standards (18). Strong predictive indexes for ICI treatment efficacy remain elusive. Pathological screening for high PD-1/PD-L1 expression is invasive and may miss diagnoses, hindering timely treatment for some patients. Non-invasive, whole-tumor-based sampling methods are urgently needed to identify immunotherapy candidates, especially for those unable to undergo surgical biopsy. Recent studies show that non-invasive imaging techniques can predict immune markers and prognosis in NSCLC patients, offering real-time assessment and survival prediction (19). Medical imaging provides objective evaluation of immunotherapy effectiveness in NSCLC. Subsequent sections explore the vital role of radiological imaging in NSCLC immunotherapy.

## Radiological imaging biomarker as a predicting immune checkpoint marker in NSCLC

### CT radiomic features predict immune checkpoint markers

Immune checkpoint markers are molecules located on the surface of immune cells, playing a pivotal role in controlling the immune response by either promoting or inhibiting immune activity (20). Specifically in cancer immunotherapy, particular checkpoint markers are employed to boost the immune system’s capability to identify and combat cancer cells. The most studied immune checkpoint markers include PD-1, PD-L1, CTLA-4, Lymphocyte Activation Gene 3 (LAG-3), T Cell Immunoglobulin and Mucin Domain-Containing Protein 3 (TIM-3), and B7 Homolog 3 (B7-H3). In clinical settings, immunohistochemistry is commonly employed for the detection of PD-L1. Nevertheless, the intricate expression of PD-L1 within the tumor microenvironment poses limitations on the comprehensive assessment of PD-L1 expression throughout the entire body using immunohistochemistry (21). CT imaging, on the other hand, not only enables the examination of macroscopic tumor characteristics but also facilitates the exploration of deeper tumor cell characteristics. CT radiomics involves the extraction of substantial medical imaging information from CT scans of patients. In addition, CT radiomics can also provide the segmentation of the tumors, feature extraction, and the establishment of models, so as to explore the molecular characteristics of the patient’s cancer cells (22, 23). TMB is known as the amounts of somatic mutations per coding area of a tumor genome. TMB has been found to serve as an important predictor of ICIs efficacy. However, the clinical availability of TMB has been limited due to challenges associated with detection, such as the need for invasive procedures followed by pathological assessment, the occasional inability to obtain adequate specimens, and the high cost of next-generation sequencing methods. Consequently, there is a pressing need to investigate alternative noninvasive biomarkers for TMB.

With the help of the combination with deep learning technology and CT radiomics (24), Montoya et al. (25) reported that radiomics features could facilitate to sorting advanced NSCLC patients received pembrolizumab for optimizing the modeling of ICI response. The predictive models incorporated baseline neutrophil-to-lymphocyte ratio and identical radiomics (surface-to-mass ratio, average Gray, and 2D kurtosis) predicted ICI response in a murine model. Through the examination of tumorous and peritumoral regions in CT images using a radiomic approach, Wu et al. (26) successfully devised a non-invasive biomarker that can effectively differentiate responders to ICIs therapy and accurately classify their survival outcomes. This biomarker has the potential to aid clinical decision-making regarding the utilization of ICIs in both advanced and resectable NSCLC patients. In the assessment of therapeutic immunotherapy, Chen et al. (27) demonstrated that CT-based radiomic models offer a non-invasive means of evaluating the presence of tumor-infiltrating CD3 and CD8 T cells in NSCLC patients. Yoon et al. (28) analyzed the correlation between morphological features and CT radiomic features of CT images of NSCLC patients and PD-L1 expression. They found that all morphological features were not correlated with PD-L1 expression (all P>0.05), while radiomic features were significantly correlated with PD-L1 expression (P=0.0007). This suggests that the use of radiomic features to evaluate PD-L1 expression status is more effective than morphological features in identifying patients who could benefit from immunotherapy. In addition, a study collected CT images of 390 patients with confirmed NSCLC and screened the 9 best radiomic features, combined with clinicopathologic risk factors to develop a prediction model of PD-L1 expression status (PD-L1 expression ≥50%) (29). The results showed that in the training group, the area under curve (AUC) of the combined model subjects was 0.829, and the AUC of the medical imaging model alone was 0.786. Therefore, the combination of clinicopathologic features can improve the prediction efficiency, and the computer-assisted automatic extraction of such features and target area outlining in the future can reduce the time invested by doctors and is expected to promote the further application of such methods. A lung cancer immunotherapy-radiomics prediction vector (LCI-RPV) with a CT radiomic-based signature was recently developed by Chen et al. (30). The authors found that this composite radiomic signature based on response vector CD274 could facilitate assessing patients’ suitability (disease response) for PD-1/PD-L1 ICIs therapy in NSCLC. Quantitative image features can be extracted from the largest primary lung tumors using CT-enhanced imaging at baseline and after the 2nd-3rd cycles of immunotherapy. The most significant features were chosen to create delta radiomics signatures, which were utilized to assess the risk stratification of patient survival following ICIs treatment. Xie et al. (31) conducted such a prediction model with pre- and posttreatment CT-based radiomics signatures, incorporating both clinicopathologic risk characteristics and phenotypic signature, to forecast progression-free survival (PFS). The integration of clinicopathologic characteristics and the delta radiomics signature within the prediction model enabled personalized prediction of PFS in ICIs-treated NSCLC patients.

Some other immune checkpoint markers were also found to be associated with CT radiomic features. It was reported that CT-based radiomics features possess the capability to predict the expression levels of CD8+ tumor-infiltrating lymphocytes (TILs) in NSCLC in the training sets (AUC= 0.83, 95% CI=0.73-0.92 vs AUC=0.68 for the test sets) (32). Wang et al. (33) proposed a non-invasive measurement technique utilizing deep learning to assess the expression of PD-L1 and predict survival outcomes in NSCLC by CT radiomics. The receiver operating characteristic AUCs were recorded at 0.950, 0.934, and 0.946, for predicting PD-L1 expression signature <1%, 1-49%, and ≥50% in the validation cohort, respectively (33). Furthermore, the study highlights that the integration of a deep learning model with clinical characteristics enhances the predictive abilities, thereby facilitating physicians in making prompt decisions regarding clinical treatment options. The above findings suggest that these radiomics features could serve as a potential medical imaging biomarker for stratifying NSCLC patients who are likely to benefit from immunotherapy, which has the potential for clinical application.

### ^18^F-FDG PET/CT predictive immune checkpoint markers

Fluor-18-deoxyglucose positron emission tomography/CT (^18^F-FDG PET/CT) is one of the most widely used molecular imaging modalities for tumor diagnosis, which is performed by utilizing glucose metabolism as a functional noninvasive medical imaging technique (34). ^18^F-FDG can be transferred to cancer cells via glucose transporter proteins, and thus be preserved in tumors and uptaken by PET. In addition, T-cell metabolism after PD-1 blockade may affect the changes in FDG uptake (35). A previous study showed that there was an association between FDG uptake and the expression of Foxp3-regulatory T cells (Tregs) in the tumor microenvironment of NSCLC (36). Lim et al. (37) confirmed that the ^18^F-FDG PET/CT radiomic model could predict the PD-L1 expression levels (AUC=0.712, sensitivity: 75.3%, and specificity: 58.2%) in patients with NSCLC. This study showed that a PET/CT-based radiomic feature can help clinicians detect positive NSCLC expressing PD-L1 in a non-invasive manner. At present, mounting studies also demonstrate that ^18^F-FDG PET/CT can be used for assessing the PD-L1 expression in NSCLC patients who underwent ICIs treatments. Silva et al. (38) implied that the whole-body total lesion glycolysis (wTLG) evaluated by ^18^F-FDG PET/CT was negatively associated with PD-L1 expression, collectively contributing to the prediction of the survival and progression to ICIs monotherapy. This study indicated that ^18^F-FDG PET/CT could effectively predict the efficacy of ICIs therapy in advanced NSCLC. Wang et al. (36) investigated the correlation between the metabolic information of ^18^F-FDG PET/CT and the expression of immune markers in patients with NSCLC. This study proved that SUVmax had a significant positive correlation with the degree of CD8+ T-cell infiltration and the expression of PD-1 and PD-L1 (P <0.05). A tumor proportional score (TPS) is calculated by comparing PD-L1-positive tumor cells to all tumor cells in the tissue sample. A previous study (39) applied TPS to determine the tumor size of untreated stage IIIB-IV NSCLC patients who underwent 18F-FDG PET/CT scanning and pulmonary lesion biopsy for PDL1 immunochemistry. The TPS was used to determine the degree of PD-L1 expression in the lung lesions of these patients, classifying the patients as having low, medium, and high PD-L1 expression (corresponding to PD-L1 TPS <1%, 1% ~49%, and ≥50%, respectively). The results showed that SUVmax could be used as a potential biomarker for PD-L1 expression and could effectively stratify the degree of PD-L1 expression in NSCLC patients. This study demonstrated that ^18^F-FDG PET/CT can facilitate to select immunotherapeutic strategy for advanced NSCLC. In addition, the combination of clinical and metabolic features showed better efficacy than metabolic features alone. Zhou et al. (40) successfully predicted NSCLC patients with high CD8+ T-cell infiltration and PD-1/PD-L1 expression in tumors by constructing a combined model based on clinical features and multiple ^18^F-FDG PET/CT metabolic features (e.g., SUVmax) (AUC = 0.869). Wu et al. (41) demonstrated that higher ^18^F-AlF-NOTA-PRGD2 (^18^F-RGD) uptake was associated with inhibited PD-L1 expression in NSCLC cells, while SUVmax was a candidate parameter to monitor tumoral expression of PD-L1. Therefore, in addition to ^18^F-FDG PET/CT, ^18^F-RGD PET/CT may be also useful for reflecting the immune status of NSCLC. However, Cui et al. (42) found that ^18^F-FDG PET/CT features could predict the pathological response after ICIs with chemotherapy in NSCLC patients, but no significant correlation was found between the radiologic response and the expression of PD-L1.

Taken together, PET/CT metabolic profiles, especially SUVmax, showed good predictive efficacy in predicting the expression level of checkpoint markers for NSCLC immunotherapy, and the combination of clinical profiles was even more effective, which could provide important information for guiding immunotherapy in NSCLC patients. However, due to the high cost of the test, its routine clinical application is limited.

## Prognostic evaluation of immunotherapy for NSCLC

### iRECIST criteria for assessing immunotherapy outcomes in NSCLC patients

The Response Evaluation Criteria in Solid Tumors 1.1 (RECIST 1.1) is a commonly used method that evaluates the response to treatment by examining changes in tumor burden on CT and MRI (43). However, there are still limitations with this criterion in evaluating abnormal immune-related tumor response patterns. Among them, the most common and challenging response pattern to medical imaging for morphologic assessment is “pseudoprogression”, i.e., the presentation of a lesion that appears to recur or become larger on medical imaging, followed by a gradual disappearance of the lesion or stabilization (44). Failure to correctly recognize pseudoprogression undoubtedly puts the patient at a higher risk of continued ineffective treatment. In order to assess this particular response pattern, a more detailed immune-response evaluation criteria for immunotherapy in solid tumors (iRECIST) was developed. With lung cancer treated with immunotherapy, iRECIST has a high inter- and intra-reader reliability, which is similar to RECIST 1.1 (45). Both RECIST and iRECIST are effective methods applied to evaluate NSCLC patients treated with ICIs or other immunotherapies (46). Though both of them are the immune-response evaluation criteria for immunotherapy in solid tumors, RECIST is primarily used to evaluate the effectiveness of conventional treatments such as chemotherapy and radiotherapy in the treatment of solid tumors, while iRECIST is apply to address immunotherapeutic approaches, such as immune checkpoint inhibitors, to evaluate their effectiveness in the treatment of solid tumors. On the other hand, RECIST evaluates the effectiveness of treatment based primarily on changes in the size of the tumor, including measurement of the diameter of the target lesion, while iRECIST can assess not only changes in tumor size, but also situations such as the emergence of new lesion that may be induced by immunotherapy, in order to more comprehensively assess treatment efficacy. Han et al. (47) demonstrated that a mixed model that combined delta-radiomics features and iRECIST had superior AUC for the major pathological response (MPR) prediction (24, 48, 49).

### CT radiomic to assess immunotherapy outcomes in NSCLC patients

In predicting and evaluating the response to immunotherapy in NSCLC patients, radiomic can provide quantitative predictors as a useful complement to RECIST-related criteria (50). Many studies indicated the application of CT radiomic in the evaluation of efficacy and prognostic assessment of NSCLC immunotherapy. CT radiomic can reflect the overall status of the tumor in a non-invasive manner. Besides, it is an effective predictor of prognosis in NSCLC patients. Texture analysis (TA) is a modern biomarker enabling the evaluation of quantitative parameters derived from various imaging modalities (e.g., CT, MRI, and Positron Emission Tomography). These parameters are associated with the distribution of pixels and voxels of gray, and they correlate with certain biological tumor characteristics, such as heterogeneity. It was reported that CT-derived texture parameters could predict the overall survival (OS) and the progression-free survival (PFS) in advanced NSCLC patients who underwent ICIs treatments (51). The mean value of positive pixels (MPP) derived from CT showed an AUC of over 70% (P < 0.001), while MPP < 56.2 was found to be significantly associated with lower OS and PFS of the patients treated with first-line immunotherapy. Yang et al. (52) retrospectively analyzed CT images of 200 advanced NSCLC patients treated with anti-PD-1/PD-L1 drugs. They classified the patients into high-risk and low-risk non-response to immunotherapy by predictive modeling prior to immunotherapy. The results showed that the PFS (11.1 months vs. 3.3 months) and OS (31.7 months vs. 17.2 months) of the low-risk group were significantly higher than those in the high-risk group. A previous study (53) utilized CT imaging characteristics and clinicopathological factors to construct a combined line plot to predict PFS in NSCLC patients treated with ICIs. The results showed that the concordance index between the predicted PFS and the actual PFS was 0.791. Therefore, constructing a prediction model by radiomic or combining it with clinical features can provide valuable information for patients’ prognosis. It is worth noting that the medical imaging radiomic models are expected to be combined with pathohistological models and immuno-scoring to further improve the prediction of immune efficacy in NSCLC patients (54).

Radiogenomics, as an emerging field of machine learning, can transform radiological images into high-dimensional data that can be mined. It helps identify specific imaging biomarkers that are associated with genetic variations related to tumorigenesis. Besides, radiogenomics can also predict the response of tumors to various treatment modalities, such as chemotherapy, radiation therapy, or immunotherapy. Using machine learning to build radiomic models to predict the survival of NSCLC patients is more effective. Liu et al. (55) used the CT-based radiomics model to predict the risk of PFS and OS in NSCLC patients treated with nivolumab. The average AUC value for predicting the PFS and OS were 73% and 0.61%, respectively. Deep learning, as a branch of machine learning, can further improve the predictive ability of radiogenomics models by learning the regular higher-order expression features of data through a large amount of computations. Tian et al. (56) established a deep learning model with significant stratification ability for PFS in NSCLC patients with different degrees of PD-L1 expression (PD-L1 expression higher than 50% is considered high-risk, while lower than 50% is considered low-risk). The PFS of NSCLC patients with different degrees of PD-L1 expression was significantly stratified (P = 0.01). The deep learning model offers a noninvasive approach for predicting elevated PD-L1 expression in NSCLC and for extrapolating clinical outcomes in the context of immunotherapy response. With the rapid development of machine learning, especially deep learning, it is expected to be more widely used in clinical applications in the future. Recent studies have shown that AI based on deep learning has been able to monitor and quantify the overall growth of tumors and discover valuable prognostic information, thus benefiting NSCLC patients treated with ICIs.

With the development of immunotherapy, the prediction of immunotherapeutic efficacy for a single tumor type has gradually increased. Several studies have confirmed that in advanced NSCLC, CT- or PET/CT-based imaging radiomic analyses are not only effective in identifying durable clinical benefit (DCB) and non-durable clinical benefit (NDB), with the AUC over 0.8 (57). Of note, combining imaging radiomic features with clinical features can further improve the ability of survival prediction. The CT-based imaging radiomic features mainly included shape-based and intensity-based features for elevating the survival prediction in lung cancer patients. Shape-based geometric characteristics of tumors in lungs include volume, surface area, sphericity, and irregularity indices. Tumors with irregular shapes or higher surface area-to-volume ratios may indicate aggressive disease and pool prognosis of the patients. The intensity-based features mean that the distribution of voxel intensities within the tumor region, i.e., mean intensity, standard deviation, skewness, and kurtosis. Deviations from normal intensity distributions indicate tumor necrosis, hypoxia, or other biological processes relevant to prognosis. Yang et al. (52) proposed a multi-omics-based deep learning approach. The authors developed a deep learning model with a simple temporal attention (SimTA) module to analyze the asynchronous clinical time series data of medical imaging histological features and laboratory indicators. They conducted a multilayer perceptron to fuse the time series coding features and static clinical information and combined the CT imaging radiomic features, laboratory examination data, and baseline clinical features to jointly construct a deep learning model. The results demonstrated that SimTA predicted that the AUC of immunotherapy for 60d was 0.77 and for the 90d efficiency was 0.8. This radiomics not only outperforms the single-omics data model but also significantly outperforms the prediction efficacy of baseline PD-L1 expression, providing a more practical model for risk stratification of advanced NSCLC patients.

## Radiological artificial intelligence predicting immunotherapy outcomes in NSCLC

A CT scan and tissue biopsy are the primary methods for diagnosing NSCLC, which can result in misdiagnosis and omissions. More and more investigators realize that non-invasive biomarkers may facilitate the diagnosis of NSCLC but should be enhanced to increase their sensitivity and specificity. Recently, radiological artificial intelligence (AI) models are proving to be an effective tool for NSCLC diagnosis, as they can improve the accuracy, stability, and efficiency of the disease (58).

Some investigators demonstrate that deep learning from AI can quantify tumorous morphological alternations, which are important for monitoring prognostication in NSCLC patients. Deep learning methodologies, specifically convolutional neural networks (CNNs), have demonstrated potential in discerning complex patterns from medical imaging data such as CT scans for prognostic assessment in NSCLC patients. CNNs have the capability to precisely delineate lung tumors and adjacent tissues from CT images, a critical process for measuring tumor characteristics such as size, shape, and spatial attributes that play a significant role in prognosis determination. These deep learning algorithms can autonomously extract intricate radiomic features from CT images, such as tumor morphology, texture, and intensity distribution, which can provide personalized prognostic estimates for NSCLC patients. Trebeschi et al. (59) employed AI to rapidly evaluate all tumor foci on pre- and post-treatment CT images of the chest of patients in a fully automated manner. From that, the authors extracted imaging radiomic features and built a radiomic model in order to predict the outcome of 152 patients with stage IV NSCLC treated with PD-1 inhibitors. The results showed that the AI-based imaging model was highly effective in predicting OS at 1 year from the date of image acquisition (AUC = 0.75). The prognostic heatmap generated by AI could also predict mediastinal lymph node metastasis and rib metastasis. Hyperprogression is the phenomenon of rapid tumor progression after immunotherapy. This paradoxical acceleration of tumor growth is found to be associated with poor prognosis. In a study by Choi et al. (60), 19.2% of patients had hyperprogressive NSCLC after ICIs treatments. A predictive model was constructed using relevant risk factors, including age, tumor volume, and metastasis to other sites. The AUC for predicting hyperprogression was as high as 0.9556 (95%CI: 0.9133-0.9978). Tunali et al. (61) enrolled 228 patients with NSCLC who were receiving single-agent or dual-agent immunotherapy. They developed a clinical-imaging radiomic predictive model based on baseline CT images and clinical information, with an AUC for predicting hyperprogression ranging from 0.804 to 0.865. Vaidya et al. (62) reviewed and analyzed the clinical and imaging data of 109 patients with advanced NSCLC treated with PD-1/PD-L1 immunosuppressant monotherapy, of whom 19 showed hyperprogression. A total of 198 radiomic features reflecting intratumoural and peritumoural textural features and quantitative vessel tortuosity around the lesion were extracted from baseline CT images. The Kaplan-Meier survival curves showed a statistically significant difference in the OS of the hyperprogression group and non-hyperprogression group predicted by the model (P<0.05). This study demonstrated that baseline CT imaging features could predict whether advanced NSCLC patients receiving immunotherapy will develop hyperprogression. Pseudoprogression refers to the appearance of new lesions or enlargement of existing lesions at the early stage of immunotherapy, while the lesions stabilize or shrink at the later stage. Therefore, pseudoprogression is not a true disease progression. Barabino et al. (63) extracted the imaging radiomic features from the baseline and the first follow-up CT of the advanced NSCLC patients. The authors showed that there were 9 differential imaging radiomic features that could identify tumor progression and pseudoprogression, while these features were not identical to those identifying tumor progression and remission. At present, there is no consensus on the mechanism and definition of the occurrence of the special response pattern of immunotherapy, thus the application value of the relevant studies needs to be further explored.

The above studies imply that AI has great potential in the evaluation of the efficacy of immunotherapy in the future, providing a new means of noninvasive individualized assessment and prediction of NSCLC patients. Nevertheless, due to the high computational requirements of AI on hardware equipment and the complexity of model design, it needs a lot of practice and verification of its effectiveness to be put into clinical application.

## Differential imaging radiomics predicting immunotherapy outcomes in NSCLC

Differential imaging radiomics is a radiological technique that calculates the changes in imaging radiomic features at different time points (e.g., pre-treatment and post-treatment) by dynamically observing these images, which can be used to analyze the correlation of treatment effects. Differential imaging radiomics enables the quantitative extraction of features from CT scans, facilitating early detection and diagnosis of NSCLC. Radiomics aids in identifying suspicious lesions indicative of NSCLC. Integration of radiomic data into prognostic models allows clinicians to effectively stratify patients based on risk of recurrence or metastasis, thereby optimizing treatment strategies. It was reported that the differential radiogenomics model was better than the baseline radiogenomics model. This novel technique was found to have a higher efficacy in predicting the presence or absence of response to immunotherapy, with AUCs ranging from 0.81 to 0.87 (64, 65). On the other hand, this differential imaging genomics model was considered to have a better predictive efficacy in adenocarcinoma than in squamous carcinoma. Khorrami et al. (66) also showed that the AUC of differential imaging radiomic features for predicting the effect of immunotherapy was above 0.8. Interestingly, the authors observed that PD-L1 expression had no significant correlation with the effect of immunotherapy. This study further found that the peritumoural regional texture feature Gabor Delta-RFs was significantly correlated with the density of tumor-infiltrating lymphocytes, suggesting that there might be a mapping relationship between the radiomic texture (DelRADx) of CT pattern and the tumor immune microenvironment. The paraneoplastic region of the tumor, where the tumor immune microenvironment is located, has an important role in the process of immunotherapy. This study suggests that we should pay attention to the information on the peritumour region to further explore its relationship with the immunotherapeutic efficacy of advanced NSCLC.

## ^18^F-FDG PET/CT assesses immunotherapy outcomes in NSCLC patients

^18^F-FDG PET/CT can also be employed to evaluate the immunotherapy outcomes in NSCLC patients. Feng et al. (67) assessed the role of ^18^F-FDG PET/CT in the clinical benefit and prognosis in patients with NSCLC. They found that metastatic lesion burden assessed by ^18^F-FDG PET/CT, such as metabolic tumor volume of primary lesion, metabolic tumor volume of whole-body, and total lesion glycolysis of whole-body, might significantly predict the response to immunotherapy in metastatic NSCLC patients (all P < 0.05). Since the molecular and functional changes of malignant tumors are more rapid than anatomical changes, tumor progression can be assessed in a timely manner at the molecular level by using ^18^F-FDG PET/CT. Mu et al. (68) extracted imaging radiomic features from PET images, CT images, and calibrated PET/CT fusion images of NSCLC patients before receiving immunotherapy. The results showed that the column maps showed excellent predictive performance, with a concordance index of 0.77 for PFS and 0.84 for OS. Valentinuzzi et al. (69) constructed an 18 F-FDG PET imaging model and compared whether it was better than the commonly used clinical criteria for predicting the response to nivolumab in patients with stage IV NSCLC. The results indicated that the imaging radiomic model was superior to the current clinical standard in predicting OS, with an AUC of 0.9 for the imaging model and an AUC of 0.79 for the clinical model. In this regard, PET/CT imaging may provide additional information beyond the clinical standard or SUV values for the efficacy and prognostic evaluation of NSCLC immunotherapy.

## Radiological imaging to identify immune-related adverse events

In addition to predicting the immunotherapeutic biomarkers and the treatment outcomes, radiological imaging is found to be associated with the identification of immune-related adverse events (IRAE) (70). Imaging genomics, an interdisciplinary field that combines radiomics with genomic data, holds great potential for predicting adverse events in NSCLC patients undergoing immunotherapy. Imaging genomics facilitates the early detection and monitoring of treatment-related toxicities by identifying imaging-genomic markers that precede clinical symptoms. Immunotherapy can lead to long-term tumor responses, which can imbalance the balance of the immune system and produce immune-related toxicity, known as IRAEs. Prediction of immune-related adverse events PD-1/PD-L1 inhibitors are often accompanied by a series of specific autoimmune toxic reactions (IRAE), caused by the recovery or enhancement of the body’s immune system function. At present, the exact mechanism of the occurrence of IRAE has not been fully clarified. IRAE commonly involves multiple systems of the organism, which usually occurs in the skin, gastrointestinal tract, lungs, and endocrine glands. Previous clinical trials have shown that nivolumab causes IRAEs in 58% of NSCLC patients and pembrolizumab causes IRAEs in 29% (71). The common IRAEs include pneumonitis, pulmonary nodulosis, pseudoprogression, hypothyroidism, diarrhea/colitis, hepatitis, and cutaneous-like reactions (72). Among them, immunotherapy-associated pneumonia is the most common IRAE in the chest.

Pneumonia is the most dangerous IRAE in patients with NSCLC. Since immunotherapy-associated pneumonia is not clinically specific and cannot be differentiated from infectious pneumonia on medical imaging, thus prevents early intervention. Currently, there are no reliable diagnostic criteria to differentiate infectious pneumonia from immunotherapy-associated pneumonia, so even mild signs, such as ground-glass shadows, atypical nodules, and reticulocytosis, should be reported as suspicious, especially in the setting of clinical deterioration. Immunotherapy-associated pneumonia often occurs within 6 months of ICIs treatment. Therefore, any imaging signs suggestive of pneumonia within 6 months of ICIs should be discussed with an oncologist and cortisone therapy should be initiated earlier. Colen et al. (73) outlined 18 regions of interest on baseline CT images of each patient, extracted a total of 1,860 medical imaging features and selected the best two features to predict the occurrence of immunotherapy-associated pneumonia with 100% accuracy. This study demonstrated that radiogenomics can be applied to stratify the risk of immunotherapy-associated pneumonia in patients before treatment. Mu et al. (74) enrolled 146 patients with advanced NSCLC, 21 of whom developed IRAE. The radiological imaging features of tumor lesions were extracted from PET images, CT images, and PET/CT fusion images. Five medical imaging features were selected to construct a radiomics score (RS), which predicted the AUC of IRAE in the different groups. Combining RS with treatment type and dosage further improved the predictive efficacy, with AUC over 0.8. This study demonstrated that higher RS, high dosage of medication, and combination of different antibodies increase the risk of IRAE occurrence. However, the sample size of IRAE in this study was still relatively small and contained multiple types of IRAE at the same time, not limited to immunotherapy-associated pneumonia. Nevertheless, the results of the study generally suggest that imaging radiomic features are closely related to the presentation of IRAE, which is instructive for subsequent studies.

In advanced NSCLC, where radiotherapy and immunotherapy are usually combined, both immunotherapy-associated pneumonia and radiation-induced pneumonitis can occur simultaneously. In that case, it is more difficult to differentiate these similar adverse events. Qiu et al. (75) outlined areas of lung injury on pulmonary window images and subsequently extracted 93 imaging radiomic features. After feature screening by the LASSO binary regression model, 11 radiomic features were employed to construct the imaging radiomic score “Rad-score”, which was able to better differentiate between immunotherapy-associated pneumonia and radiation-induced pneumonia with an AUC of 0.89. The Rad-score combined with clinical features (bilateral changes and sharp borders) had an AUC of more than 0.95 for differentiating the two types of pneumonia. Three phase 3 trials (including Impower 130, 132, and 150 studies) revealed that the OS of patients with IRAEs was significantly longer than that of patients without IRAE in both the immunotherapy and control groups (76). This study suggests that patients with IRAEs tend to be the beneficiaries of ICIs treatment, while patients without IRAEs may be ineffective for ICIs treatment. Thus, IRAEs can be used as a hallmark event of treatment benefit in NSCLC patients and prompt timely clinical intervention. On the other hand, clinicians need to identify whether the treatment is effective in patients without IRAEs and take countermeasures in a timely manner. In recent years, as ICIs become more widely used in clinical practice, there is a need for medical imaging physicians and clinicians to utilize imaging to identify IRAEs in a timely manner and to treat them early in order to improve the survival of NSCLC patients.

## The underlying molecular mechanisms of the potential of radiomics in NSCLC

CT-based radiomics plays a significant role in NSCLC by providing insights into the molecular characteristics of tumors through the analysis of medical images. NSCLC is known for its molecular heterogeneity, indicating tumors can differ greatly in their genetic and molecular profiles. Radiomics aims to capture this heterogeneity non-invasively by correlating imaging features with molecular markers such as protein expression (e.g., PD-L1), genetic mutations (e.g., EGFR mutations), or metabolic activity (e.g., glucose metabolism). The variability in tumor tissue infiltration and fluctuations in PD-1 expression over time and space present challenges in the accurate and consistent detection and assessment of PD-1+ tumor-infiltrating lymphocytes (TILs). Mierzwicka et al. showed that PD-1-targeted small protein variants for *in vivo* PET imaging in NSCLC. It was reported that β-sheet-derived MBA variants exhibited PD-1 specificity and stability, which was sufficient for sensitive *in vivo* PET/CT imaging. The distribution of 68Ga-MBA proteins in mice was monitored using whole-body positron emission tomography combined with PET/CT imaging. Mierzwicka et al. (77) found that MBA proteins demonstrated strong binding affinity and specificity towards human and mouse PD-1 receptors, whether in recombinant form or on the surface of cells, offering a viable non-immunoglobulin alternative for visualizing PD-1+ cells in PET imaging. As such, MBA proteins have the potential to serve as valuable diagnostic tools for guiding the clinical care of patients with NSCLC under immunotherapy (77). Radiomics is an emerging method that can convert medical images into quantitative data to profile tumor phenotypes. Tian et al. (56) evaluated the PD-L1 expression in NSCLC and predicted the therapeutic responses to ICIs by applying deep learning on CT images. PD-L1 expression signature could predict the high level of PD-L1 (PD-L1 expression≥ 50%) with an AUC of 0.78 (95% CI: 0.75 to 0.80) (56).

Previous study showed that EGFR mutation was an effective target for improving the overall survival in patients with advanced NSCLC (78). EGFR plays significant biological roles in NSCLC patients receiving immunotherapy by modulating the immune microenvironment and influencing tumor-infiltrating lymphocytes. A biological rationale implied that sensitivity to ICIs was higher in tumors bearing high levels of EGFR mutations (79). However, NSCLC tumors with EGFR mutations (such as exon 19 deletions or L858R mutations) traditionally respond poorly to ICIs targeting PD-1/PD-L1 (80). This is because these mutations often lead to lower tumor mutational burden and reduced expression of neoantigens, which are targets for immune recognition and activation. Based on this evidence, recent researches rarely focus on EGFR-targeted therapy in NSCLC.

In the view of glucose metabolism, Tricarico et al. (81) investigated the metabolic tumor volume in patients with metastatic NSCLC underwent immunotherapy by assessing 18F-fluoro-deoxy-glucose positronemission tomography (18F-FDG PET/CT). It is known that 18F-FDG PET metabolic parameters (i.e., tumor maximum standardized uptake value and metabolic tumor volume) are correlated to the outcome of immune checkpoint inhibitors in NSCLC (82). This study demonstrated that 18F-FDG PET/CT volume-based metrics were the effective prognostic non-invasive biomarkers in patients with advanced NSCLC underwent immunotherapy treatments (81). However, a previous study developed by Puyalto et al. (83) indicated that conventional 18F-FDG-PET scans could not detect the antitumor activity in immunotherapy-treated patients. Nevertheless, the authors found that [89Zr]-anti-PD-1 uptake was significantly elevated in mice that responded to PD-1 blockade. Besides, they also identified a positive correlation between [89Zr]-anti-PD-1 uptake and the level of TILs (r = 0.8; P= 0.001).

## Comparisons of radiomics with other emerging biomarkers and diagnostic tools

In the evaluation of immunotherapeutic outcomes in NSCLC, both radiomics and other biomarkers like PD-L1 expression and tumor mutation burden (TMB) play crucial roles (**Table 1**). Radiomics can potentially predict immune checkpoint marker expression indirectly by capturing tumor heterogeneity, shape, and texture features that correlate with the tumor microenvironment. Previous studies showed that radiomic features might correlate with areas of immune infiltration or spatial patterns indicative of immune checkpoint expression (84, 85). Yang et al. (86) demonstrated that a high radiomics score was associated with the related signaling pathways suppressing tumor proliferation and the infiltration of antitumor immune cell in NSCLC. Tumor-infiltrating lymphocytes (TIL) are the reliable biomarker of ICIs in NSCLC. Park et al. (87) established a CT radiomics model by extracting the radiomic features in NSCLC. They found that enrichment of TILs was dramatically correlated to the PFS independent of PD-L1 status [4]. Though radiomics offers non-invasive and comprehensive insights into tumor characteristics, several limitations should be acknowledged, i.e., lack of standardization, uncertain data quality, and requirements of clinical validation. While the above biomarkers (e.g., PD-L1, TMB, and TIL) directly reflect tumor biology and its interaction with the immune system, which are already incorporated into clinical practice for guiding immunotherapy decisions. However, some biomarkers require invasive tissue sampling (biopsy), which may not always be feasible or desirable. Besides, biomarker levels can vary spatially within a tumor and over time, potentially leading to sampling bias or incomplete assessment. Also, they may not fully capture tumor heterogeneity or other complexities that influence treatment response. Based on these facts, radiomics and biomarkers can provide complementary information about tumors. Combining radiomic features with molecular biomarkers could enhance predictive accuracy in the field of NSCLC researches, offering a more personalized therapeutic approach.

**Table 1:** The characteristics of the included studies reporting the application of CT and PET/CT-based radiomics in predicting and evaluating immunotherapy responses in NSCLC patients.

Study and references	Study area	Study population (n)	Mean age (years)	NSCLC stage	Imaging modality	Radiomic features	Outcomes (HR, 95%CI, AUC, or P value)
Arthur (23) 2023	UK	259	59	phase 3	CT-based radiomics	Manual delineation, sub-segmentation, feature extraction, and predictive model building	Differentiated liposarcoma from leiomyosarcoma with an AUROC of 0.928; Predicted histological grade with an AUROC of 0.882 on validation.
He (24) 2020	China	327	61.8 ± 10.2	stage IV (85.4%)	TMB radiomic biomarker for predicting immunotherapy efficacy	CT images from 327 patients with TMB data; Applying 3D-densenet to estimate the target tumor area, which used deep learning features and establish the TMB radiomic biomarker.	High-TMB and Low-TMB patients in the training cohort (AUC: 0.85, 95% CI: 0.84 to 0.87) and test cohort (AUC: 0.81, 95% CI: 0.77 to 0.85). TMB radiomic biomarker divided patients into a high- and low-risk group with distinctly different OS (HR: 0.54, 95% CI: 0.31 to 0.95; p=0.00) and PFS (HR: 1.78, 95% CI: 1.07 to 2.95; p=0.023).
Montoya (25) 2023	USA	117	67	Stage IV	CT-based radiomics	Surface-to-mass ratio, average Gray value, and 2D kurtosis.	The average of the AUCs yielded an AUC of 0.75. Radiomics features were associated with ICI response in 2 independent cohorts.
Wu (26) 20	China	319	58.7± (12.5)	Stage III-IVB	CT-based radiomics	Picture Archiving and Communication System, contrast-enhanced thoracic CT. Generated 3D volumes of interest (VOIs) of tumors	The combined radiomic signature (CRS) in predicting immunotherapy responder was better than PD-L1 expression (AUC: CRS=0.82, 95% CI: 0.75 to 0.88; PD-L1 = 95% CI: 0.50 to 0.69).
Chen (27) 2023	China	105	65.18 ± 8.78	Stage I-IV	CT-based radiomics	Extracting 1316 texture-based radiomics features. The features acquired after LASSO regression. Radiomics model was also based on CD3 and CD8 expression.	CD3 radiomics model has an AUC of 0.943 (95% CI: 0.886-1), while the AUC of the CD8 radiomics model was 0.837 (95% CI: 0.745-0.93). Patients with high expression of CD3 and CD8 had better radiographic results than those with low expression (p<0.05).
Yoon (28) 2020	Korea	153	64.1 ± 11.2	stage >IIIA	CT-based radiomics	The prediction models were established by multivariate logistic regression analysis, combining of clinical variables and radiomic features.	The predictive performance on predicting PD-L1 positivity was higher with model of combination of clinical variables and CT radiomic features (c-statistic = 0.667; 95% CI = 0.575–0.760) than the model with only clinical variables (c-statistic = 0.550; 95% CI = 0.454–0.646).
Sun (29) 2020	China	390	62.31 ± 10.55	Stage I-IV	CT-based radiomics	Based ono the in-house texture analysis software with algorithms, radiomic features extracted from the tumor ROI. 9 out of 200 radiomic features were selected to develop radiomics signature.	PD-L1 expression of NSCLC tumors was significantly associated with radiomics signature. For prediction of PD-L1 expression, the prediction model that combination of radiomics signature and clinicopathologic features for predicting PD-L1 expression resulted in AUC 0.848 in the validation cohort.
Chen (30) 2023	China	194	68.2 ± 9.2	Stage I-IV	Radiogenomics Biomarker	Radiomic features extracted from segmented tumors on contrast-enhanced chest CTs. For predicting PD-L1, an immunotherapy- radiomics prediction vector was applied.	Immunotherapy-radiomics prediction vector (IRPV) could predict PD-L1 positivity in NSCLC testing cohort (AUC = 0.7, 95% CI: 0.57-0.84). IRPV could also stratify patients into a high- and low-risk survival group (HR= 2.26, 95% CI: 1.21-4.24, p = 0.011 and HR = 2.45, 95% CI: 1.07-5.65, p = 0.035).
Xie (31) 2022	China	97	NA	Stage I-IV	CT-based radiomics	A prediction model combining the clinicopathologic risk characteristics and phenotypic signature.	The C-index of radiomics models in the validation cohort was 0.78. The radiomics score showed good accuracy for distinguishing patients with progression to ICI treatment. The delta radiomics model had a significant higher predictive accuracy compared to PD-L1 expression (P<0.0001).
Chen (32) 2023	China	117	59.09 ± 11.8	Stage I-IV	CT-based radiomics	LASSO regression was applied to evaluate strongest features for abundance of CD8+ TILs, which was used to construct the radiomics score.	The radiomics score was correlated to an abundance of CD8+ TILs in NSCLC (AUC=0.83, 95% CI=0.73-0.92) in the training set, while the AUC in the test set was 0.68 (95% CI=0.54-0.87).
Wang (33) 2022	China	1135	58.77 ± 10.66	Stage I-IV	CT-based radiomics combined with deep learning	The deep learning feature was obtained through a 3D ResNet, while radiomics were based on CT images.	The combination AI model showed a high-performance with AUCs of 0.95, 0.934, and 0.946 for predicting PD-L1 expression signature <1%, 1-49%, and ≥50%, respectively. The combination AI model was trained on multi-source features the performance of OS evaluation in NSCLC patients (C-index: 0.89).
Wang (36) 2020	China	763	> 60 (419/763)	Stage I-IV	18F-FDG-PET/CT	Patient image data were acquired applying a DiscoveryST4 PET/CT scanner.	SUVmax on 18F-FDG PET/CT was an independent prognostic factor in NSCLC patients (p = 0.013). SUVmax was significantly associated with the expression of CD8 tumor-infiltrating lymphocytes, CD163 tumor-associated macrophages, Foxp3-regulatory T cells, as PD-1 and PD-L1 (all P<0.05).
Lim (37) 2022	Korea	312	66.2 ± 9.1	Stage I-IV	PET/CT-based Radiomics	PET radiomic features were extracted from segmented tumors on PET and CT images using the LIFEx package. The top five best feature subsets were chosen through the Gini index.	For predicting PD-L1 expression, the AUC of the Naïve Bayes model based on PET/CT was 0.712, with a sensitivity of 75.3% and specificity of 58.2%.
Silva (38) 2022	Brazil	98	NA	Metastatic or IIIC	18F-FDG PET/CT	Radiomics based on maximum standardized uptake values, whole metabolic tumor volume (wMTV), as well as whole-body total lesion glycolysis (wTLG).	Lower baseline 18F-FDG PET/CT parameters were correlated to the responses to ICIs. Patients with higher wTLG were correlated to the progression and worse survival to ICIs therapy.
Wang (39) 2020	China	133	64 (median)	Stage IIIB-IV	18F-FDG PET/CT	Radiomics based on the mean and maximum SUV, metabolic tumor volume, and total lesion glycolysis of primary lesions.	Mean (pSUVmean) and maximum standard uptake values (pSUVmax) were increased with the increase of pulmonary lesions PD-L1 expressions, while pSUVmax rather than pSUVmean was an independent predictor for pulmonary lesions PDL1 (odds ratio= 4.82, 3.92, and 4.45 for pPDL1-negative,-moderate, and -strong, respectively).
Zhou (40) 2021	China	91	59 (36~78)	Stage I-IV	18F-FDG PET/CT	PET images were attenuation-corrected and anatomically fused with low-dose CT images. SUVmax, SUVmean, and metabolic tumor volume were measured.	dSUVmax, dSUVmean, eSUVmax, eSUVmean, ΔSUVmax, ΔSUVmean, and ΔTLG were significantly higher in patients with positive PD-L1. High glucose metabolism on dual-time-point 18F-FDG PET correlated with the tumor microenvironment immune types-I tumors.
Wu (41) 2022	China	30	NA	Stage III-IV	18F-RGD	PET/CT images were viewed using a Xeleris workstation. The analysis was assisted with the MIM software package.	The SUVmax was significantly negatively associated with tumor PD-L1 expression (P=0.014). Higher 18F-RGD uptake was associated with depressed PD-L1 expression.
Cui (42) 2022	China	30	59 (33-71)	Stage III	18F-FDG PET-based radiomics	Most of radiomics features (76/102) were highly reproducible with ICCs higher than 0.75.	Twenty patients (20/30) achieved major pathological response and 16 of them (16/30) achieved complete pathological response. SUVmax, SUVpeak, SULpeak, and End-PET-GLDM-Large Dependence High Gray Level Emphasis (End-GLDM-LDHGLE) were independently associated with complete pathological response.
Huicochea (45) 2021	USA	85	69 (41–92)	NA	CT scans	iRECIST and RECIST 1.1 based on CT scans	Patients with lung cancer treated with immunotherapy exhibit high inter- and intra-reader agreement on iRECIST.
Singla (46) 2021	India	28	59.3 (27-78)	metastatic NSCLC	CT scans	RECIST 1.1 and iRECIST	iRECIST could monitor treatment more precisely. Meanwhile, it could also weight against infrequent pseudoprogression of NSCLC.
Han (47) 2024	China	206	61 ± 6.6	Stage IIA-IIIB	Contrast-enhanced CT scans	The delta-radiomics features were characterized by radiomics features between baseline and preoperative.	For predicting the major pathological response, delta-radiomics model showed the AUCs values of 0.716 in the two external validation databases.
Mu (48) 2021	USA	697	62.71 ± 8.78	Stage I-IV	Deep learning of 18F-FDG-PET/CT	18F-FDG-PET/CT images were analyzed using a SResCNN to develop a deeply learned score (DLS).	DLS significantly discriminated between PD-L1 negative and positive patients (all AUC ≥0.8 in three cohorts). DLS combined with clinical characteristics could accurately predict the survival of the NSCLC patients (C-indexes of 0.70-0.87).
He (24) 2020	China	123	61.8 ± 10.2	Stage III-IV	TMB radiomic biomarker (TMBRB)	Based on feature extraction and classification module, patient’s tumor areas were set as training data to identify patients with High-TMB.	TMBRB could distinguish between High-TMB and Low-TMB patients in both training cohort (AUC= 0.85, 95% CI: 0.84 - 0.87) and test cohort (AUC= 0.81, 95% CI: 0.77 - 0.85). The predictive value of TMBRB was superior than that of a radiomic model or histological subtype.
Park (49) 2020	Korea	29	64 (median)	NA	FDG-PET/CT	A deep learning model to predict cytolytic activity score (CytAct) using segmented tumors on FDG-PET.	The predictive model of CytAct was associated with tumor size after ICB treatment (r = -0.54, p < 0.001). It was also associated with the survival of the NSCLC patients (HR =0.25, p = 0.001 and 0.18, p = 0.004, for PFS and OS, respectively).
Zerunian (51) 2021	Italy	21	59 (45-82)	Advanced NSCLC	CT-based radiomics	After obtaining the volumetric region of interests, radiomic features were extrapolated using the image histogram statistical method.	Among texture parameters, mean value of positive pixels (MMP) showed the AUC over 70% in patients with advanced NSCLC with Pembrolizumab treatment (P < 0.001). MPP was significantly associated with both OS and PFS (all P < 0.05).
Yang (52) 2021	China	200	62 (61-64)	Advanced stage NSCLC	Multi-omics-based serial deep learning on CT scans	The radiomic features were extracted with PyRadiomics for deep learning.	The predicting model exhibited a good performance in distinguishing responders from non-responders with anti-PD-1/PD-L1 treatment, with an AUC of 0.8 (95% CI: 0.74-0.86).
Yang (53) 2021	China	92	NA	Stage I-IV	CT-based radiomics	Radiomics model was constructed based on the Rad-score.	The AUC values for the training and validation cohorts were 0.848 and 0.795 (all P<0.05). Combined with CT-based radiomic features and clinicopathological factors could be used prior to the initiation of immunotherapy.
Liu (55) 2021	China	46	62 (46–77)	Stage IIIB-IV	CT-based radiomics	The CT scans were reconstructed through the standard convolution kernel.	The average HR of the models for predicting OS and PFS were 3.54 and 6.22 (p<0.05) in patients received nivolumab. The AUC for this model was 0.73.
Tian (56) 2021	China	939	58.8 ± 10.7	Stage IIIB-IV	Deep learning on CT images	Deep learning features were primarily extracted from the CT tumor slice input.	This deep learning model could predict high PD-L1 expression of NSCLC (all AUC>0.70 in three cohorts) and to infer clinical outcomes in response to immunotherapy. Low PD-L1 expression signature was associated with improved PFS (HR= 2.57, 95% CI: 1.22~5.44; P = 0.010).
Zhou (57) 2023	China	94	83.8% <75	Stage IIIB-IV	Deep learning on multiparameter prediction model and CT images	A radiomics-based CT (first order, shape, texture, and wavelet) was generated by using the SlicerRadiomics package.	The AUCs in the three cohorts (the training set, cross-validation set, and external validation set of the patients with chemo-immunotherapy) based on the principal components analysis (PCA) and support vector machine (SVM) were all over 0.7.
Tunali (61) 2019	Turkey	228	62 (61-64)	NA	Image-based features (radiomics) on CT scans	Based on Synthetic Minority Oversampling Technique (SMOTE).	The parsimonious clinical-radiomic models with modest to high ability to predict rapid disease progression phenotypes with AUC over 0.7. For survival outcomes, atients who had time-to-progression< 2 months were based on patient-level probabilities.
Vaidya (62) 2020	USA	109	NA	Stage I-IV	CT-based radiomics	CT scans pretreatment were analyzed to examine radiomic texture patterns within and around the target nodules, and to determine tortuosity of the nodule’s associated vasculature.	Radiomic features could predict the hyperprogression in patients with ICB treatments with an AUC of 0.85 in the training set and 0.96 in the validation set.
Liu (64) 2021	China	197	63 (35–84)	NA	CT-based radiomics	Delta-radiomics feature based on the relative net change in radiomics feature between baseline and first follow-up.	Delta-radiomics nomograms incorporating Delta-radiomics signatures with clinical factors of distant metastasis for target lesions provided satisfactory performance in identifying responders underwent anti-PD1 immunotherapy with an AUC of 0.83 (95% CI: 0.75-0.91).
Gong (65) 2022	China	224	65 (27–86)	Stage III-IV	CT-based radiomics	CT-based radiomics were calculated by segmenting tumors, resampling images, identifying features, and normalizing them	The rad-score of delta-radiomics model showed a significant prognostic for PFS and OS in validation cohorts in patients with immunotherapy (P < 0.05).
Khorrami (66) 2020	USA	139	65 (42–83)	Distant metastasis	CT-based radiomics	Based on a machine learning setting, the radiomic texture (DelRADx) of CT patterns both within and outside tumor nodules was established.	DelRADx features yielded an AUC of 0.88 ± 0.09. The response prediction accuracy for the DelRADx was 88% for patients treated with nivolumab. Besides, DelRADx features were also associated with the OS in patients underwent immunotherapy.
Feng (67) 2023	China	34	>60 (22/34, 65%)	Stage III-IV	18 F-FDG PET/CT	To reconstruct images from PET/CT whole-body images, the ordered subset maximum expected value method is employed (OS-EM).	Patients with advanced NSCLC with a high amount of metastatic lesions can be predicted to respond to immunotherapy based on 18 F-FDG PET/CT scans.
Mu (68) 2020	USA	194	66.76 ± 13.64	Stage IIIB-IV	Radiomics of 18F-FDG PET/CT	Based on minimum Kullback-Leibler divergence (KLD) criteria, the radiomic features from PET, CT, and PET+CT fusion images were extracted.	The multiparametric radiomics signature (mpRS) could predict patients who might receive durable clinical benefit, with the AUC of 0.81 (95%CI 0.68-0.92) in the prospective test cohorts.
Valentinuzzi (69) 2020	Slovenia	30	65 (46–77)	Metastatic NSCLC	18F-FDG PET/CT	FDG radiomics features were extracted from primary tumors, including volume, SUVmax, and SUVtotal.	The baseline radiomics features could predict the response of metastatic NSCLC patients treated with pembrolizumab HR = 0.46, p = 0.007; AUC = 0.85, 95% CI 0.69-1.00).
Mu (74) 2020	USA	146	69.43 ± 6.72	Stage IIIB-IV	Radiomics of 18F-FDG PET/CT	Radiomics features were extracted from baseline PET, CT, and PET/CT fusion images to generate a radiomics score (RS) to quantify patient risk for developing immune-related adverse events (irSAEs).	The radiomics nomogram, incorporating the RS, type of immune checkpoint blockade, and dosing schedule, was able to predict patients with and without irSAEs with area under the receiver operating characteristic curve of 0.92 (95% confidence interval [CI]: 0.86, 0.98), 0.92 (95% CI: 0.86, 0.99), and 0.88 (95% CI: 0.78, 0.97) in the training, test, and prospective validation cohorts,
Qiu (75) 2022	China	126	57.1	NA	CT-based radiomics	A logistic regression was used to establish a radiomics nomogram.	The radiomics nomogram model differentiated between checkpoint inhibitor-related pneumonitis and radiation pneumonitis with the empirical and α-binormal-based AUCs of 0.891 and 0.896.
Tricarico (81) 2024	France	110	63 (39–91)	Stage IV	Radiomics of 18F-FDG PET/CT	Based on the features of 18F-FDG PET/CT, SUVmax, total metabolic tumor volume (TMTV), total lesion glycolysis (TLG) were calculated.	TMTV was predictive of OS (AUC =0.64; 95% CI: 0.61 to 0.66). TMTV1 prognostic stratification was independent of PERCIST criteria on both PFS and OS.
Seban (82) 2020	France	80	61.9 (34.2–84.8)	Advanced NSCLC	Radiomics of 18F-FDG PET/CT	Based on radiomics 18F-FDG PET/CT, total metabolic tumor volume (TMTV) was evaluated.	TMTV > 75 cm3 was associated with shorter OS (HR= 2.5, 95%CI: 1.3-4.7) for ICI treatment in advanced NSCLC patients.
Huang (84) 2016	China	282	61 (21–84)	Stage IA-IIB	CT-based radiomics	A radiomics signature was established by the least absolute shrinkage and selection operator, or LASSO, Cox regression model.	The radiomics-based nomogram showed a better performance for predicting DFS (C-index: 0.72; 95% CI: 0.71 to 0.73) than with the clinical-pathologic nomogram.
Yang (86) 2024	China	185	61.95 ± 8.65	Stage I-IV	Radiomics of 18F-FDG PET/CT	Radiomics models were established via the LASSO method.	The ability of PET-CT radiomics model to predict pathological complete response with an AUC of 0.818 (95% CI: 0.711, 0.925).
Park (87) 2022	Korea	220	≥60 (64.1%)	Stage I-IV	CT-based radiomics	A LASSO model was constructed using features that was associated with tumor-infiltrating lymphocytes (TIL) enrichment.	The patients with high predicted TIL had significantly prolonged PFS compared to those with low predicted TIL (median: 4.0 months, 95% CI: 2.2-5.7).

NA, Not available; AUROC, Area under the receiver operator curve; TMB, tumor mutational burden; HR, Hazard ratio; CI, Confidence interval; OS, Overall Survival; PFS, Progress Free Survival; LASSO, Least absolute shrinkage and selection operator, AI, Artificial intelligence; TMB, Tumor mutational burden; ICB, immune checkpoint blockade.

As the present knowledges of AI and machine learning for predicting the outcomes of immunotherapy in NSCLC, the quality of medical imaging data may be affected by a variety of factors, such as differences in imaging equipment, inconsistencies in scanning parameters, and individual patient differences, which may lead to data bias and affect the accuracy and reliability of the model. Also, different individuals with different immunotherapeutic agents may have unique imaging characteristics, leading to poor performance and limited generalisability of established models. In addition, lung tumorous change during immunotherapy are dynamic. At present, however, the existing radiological imaging, AI, or machine learning methods still need to be improved in their ability to monitor and predict efficacy in real-time and continuously. Also, it should be noted that running complex AI algorithms usually requires significant computational resources, which may be difficult to meet in some healthcare departments, limiting their practical application. Based on the above evidence, although radiological imaging-related AI and machine learning have great potential for predicting immunotherapy efficacy in NSCLC, further research and improvements are needed to overcome the current shortcomings.

## Limitations and challenges

Implementing radiomics and AI in patients with NSCLC underwent immunotherapy presents several challenges and limitations. As aforementioned, radiomics relies heavily on imaging data, which must be of high quality and standardized across different institutions. Variations in imaging protocols, equipment, and techniques can affect the reliability and reproducibility of radiomic features, leading to inconsistent results. Besides, identifying relevant radiomic features associated with immune checkpoint markers and immunotherapy outcomes requires rigorous selection and validation processes. Overfitting and selection bias are common challenges when dealing with high-dimensional radiomic data, which can lead to unreliable predictions. At present, since underlying biological mechanisms linking radiomic features extracted from imaging data to immune responses and treatment responses are often complex, there is still a need to establish strong biological and clinical correlations between radiomic features and immune-related biomarkers (such as PD-L1 expression) or treatment outcomes. As the accessibility, while AI and radiomics can generate predictive models, the interpretability of these models remains a significant hurdle. Finally, implementing radiomics and AI may require significant investment in terms of technology, expertise, and training. This can pose barriers, particularly for smaller healthcare institutions or those with limited resources. Addressing these challenges and limitations will be crucial for harnessing the full potential of radiomics and AI in optimizing immune checkpoint marker assessment and predicting immunotherapeutic outcomes in NSCLC. In future, it is essential to overcome the aforementioned obstacles the field towards personalized medicine in NSCLC treatment.

## Summary and outlook

**Figure 1:**
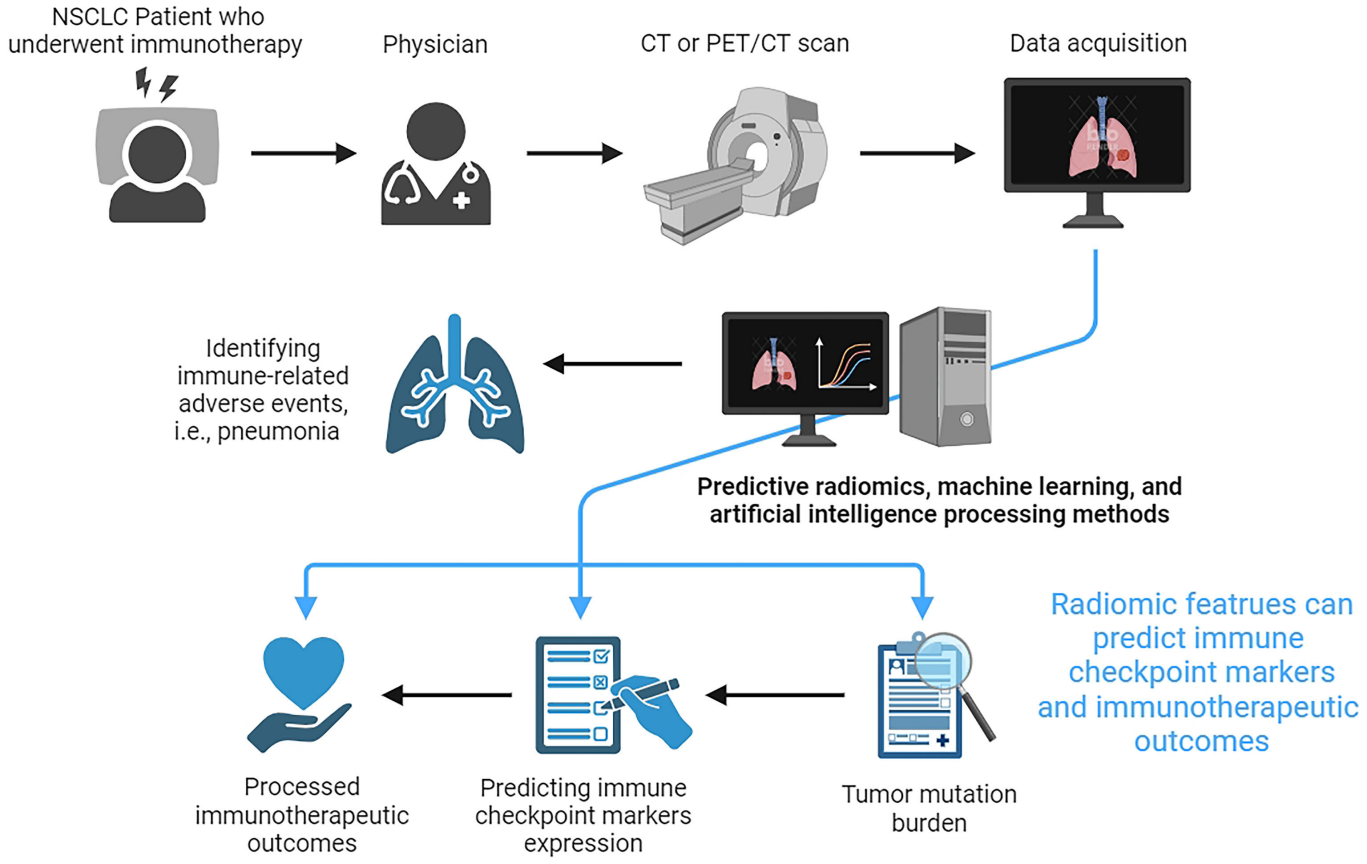
The workflow for using CT-based radiomics to predict the expression of immune checkpoint markers, immunotherapeutic outcomes, and immune-related adverse events in non-small cell lung cancer.

The early prediction and the treatment outcome assessments in advanced NSCLC patients who underwent immunotherapy are crucial for improving patients’ prognosis. Relevant studies demonstrate that radiomics is effective in predicting the expression of immune checkpoint markers and the effect of immunotherapy in a non-invasive, real-time, and dynamic way, which has a broad application prospect in the future clinical diagnosis and treatment of NSCLC. **Figure 1** shows the workflow for using CT-based radiomics to predict the expression of immune checkpoint markers, immunotherapeutic outcomes, and immune-related adverse events in NSCLC. However, some inherent limitations should be acknowledged when applying medical imaging techniques for immunotherapy of NSCLC. First, medical imaging models have been developed for diagnostic and therapeutic evaluations or prognostic judgments, but the true significance of such studies has occasionally been questioned due to the lack of logic inherent in the computerized processing of medical imaging information. Second, the reproducibility of medical imaging models is still controversial. There is still a choke point of poor consistency between different algorithms. Third, immunoimaging often relies on novel molecular probes, most of which are in the animal stage. Therefore, the clinical safety and efficacy of these novel techniques have yet to be verified. Finally, in addition to PD-L1, there are many other checkpoint markers for subtypes of lung cancer. How to use advanced imaging technology to predict these new antigens and evaluate the efficacy and prognosis of immunotherapy by using these antigens needs to be further explored. Reassuringly, with the rapid development of medical imaging AI and machine learning, the further integration of multi-omics data, and the deeper enhancement of algorithms, radiological imaging is expected to provide strong support for the development and selection of clinically individualized treatment plans for NSCLC patients who received immunotherapy.
